# Hesperetin attenuates the expression of markers of adipose tissue fibrosis in pre-adipocytes

**DOI:** 10.1186/s12906-023-04152-z

**Published:** 2023-09-11

**Authors:** Alemeh Taheri, Samira Ezzati Mobaser, Pegah Golpour, Mona Nourbakhsh, Masoumeh Tavakoli-Yaraki, Sahar Yarahmadi, Mitra Nourbakhsh

**Affiliations:** 1 Department of Biochemistry, School of Medicine, Iran University of Medical Sciences, Hemmat Highway, Tehran, 1449614535 Iran; 2 Metabolic Disorders Research Center, Endocrinology and Metabolism Molecular- Cellular Sciences Institute, Tehran University of Medical Sciences, Tehran, Iran; 3 Department of Biochemistry, School of Medicine, Shahid Sadoughi University of Medical Sciences, Yazd, Iran; 4 Hazrat Aliasghar Children Hospital, School of Medicine, Iran University of Medical Sciences, Tehran, Iran; 5 Finetech in Medicine Research Center, Iran University of Medical Sciences, Tehran, Iran

**Keywords:** Obesity, Adipose tissue fibrosis, Hesperetin, Collagen, Osteopontin, Matrix metalloproteinase

## Abstract

**Background:**

Excessive extracellular matrix (ECM) deposition in adipose tissue is a hallmark of fibrosis, leading to disrupted adipose tissue homeostasis and metabolic dysfunction. Hesperetin, a flavonoid compound, has shown promising anti-inflammatory, anti-obesity and anti-diabetic properties. Therefore, we investigated the anti-fibrotic effects of hesperetin, through targeting ECM components and matrix metalloproteinase enzymes.

**Methods:**

3T3-L1 cells were cultured in DMEM, containing 10% FBS and 1% penicillin/streptomycin. Cells were treated with a range of hesperetin concentrations, and the cell viability was determined using MTT assay. Subsequently, the expression of genes encoding collagen VI, osteopontin, matrix metalloproteinase-2 (Mmp-2) and Mmp-9 was analyzed using specific primers and real-time PCR technique. To evaluate protein levels of collagen VI and osteopontin, Western blotting was performed.

**Results:**

Hesperetin affected the viability of 3T3-L1 adipocytes with IC50 of 447.4 µM, 339.2 µM and 258.8 µM (24 h, 48 and 72 h, respectively). Hesperetin significantly reduced the gene and protein expression of both collagen VI and osteopontin in 3T3-L1 pre-adipocytes, in a time- and dose-dependent manner. Hesperetin was also able to cause a remarkable decline in gene expression of Mmp2 and Mmp9.

**Conclusion:**

Hesperetin could potently reduce the production of markers of adipose tissue fibrosis and might be considered a potential anti-fibrotic compound in obesity. Thus, hesperetin has the potency to be used for the treatment of obesity-associated fibrosis.

**Supplementary Information:**

The online version contains supplementary material available at 10.1186/s12906-023-04152-z.

## Introduction

Over the past decades, there has been a dramatic increase in global obesity prevalence [1], and according to WHO’s estimate, it will increase by roughly 167 million cases by 2025 (www.who.int). Obesity is associated with increased mortality and numerous complications including type 2 diabetes mellitus (T2DM), non-alcoholic fatty liver disease (NAFLD), non-alcoholic steatohepatitis (NASH), cardiovascular disease and a number of cancers [2, 3]. The progression of obesity is closely associated with maladaptive adipose tissue remodeling in response to excess calorie intake. This phenomenon is characterized by adipocyte hypertrophy and/or hyperplasia, which lead to hypoxic conditions and continuous production and deposition of extracellular matrix (ECM) [4]. The matrix itself is capable of promoting differentiation of progenitor cells into adipocytes to accommodate adipose tissue expansion and further fat storage. This excessive deposition of potentially pathological ECM components is defined as adipose tissue fibrosis [5]. Adipose tissue fibrosis has a complex and crucial role in obesity-related metabolic dysfunction. Increased ECM in adipose tissue causes adipocyte death, reduced lipolysis, and disturbed cell-cell interactions [6]. Notably, adipose tissue fibrosis is linked to insulin resistance in subjects with obesity [7, 8]. Inhibition of adipose tissue fibrosis has been shown to be an effective mechanism to improve glucose homeostasis, so targeting adipose tissue fibrosis has been suggested as an efficient tool to alleviate insulin resistance in obesity [9].

Collagen VI is a highly enriched ECM component of adipose tissue and is specific to adipose tissue fibrosis. The removal of collagen VI in knockout mice models of obesity resulted in uninhibited expansion of adipocytes and was accompanied by considerable improvements in lipid clearance, pancreatic hyperplasia, insulin function, and whole-body energy homeostasis, while lower inflammation and necrotic cell death occurred [10]. Moreover, endotrophin which is a component of collagen VI, stimulates fibrosis and inflammation and eventually leads to increased insulin resistance [11].

Osteopontin, a multifunctional ECM-associated protein, is produced by adipose tissue and is significantly elevated in visceral adipose tissue in obesity [12]. Enhancement of osteopontin has been shown to be associated with adipose tissue inflammation and insulin resistance [13]. Deletion of osteopontin was shown to lead to reduced adipose tissue fibrosis and ECM remodeling, reduced MMP2 and MMP9 activity, followed by higher body temperature, improved brown adipose tissue function, reduced body weight and fat mass, and higher insulin sensitivity [14].

Hesperetin, a natural phenolic compound, belonging to flavanone class of flavonoids and an aglycon of hesperidin, is present in citrus fruits such as oranges and grapefruit [15]. It possesses a number of health benefits and exerts anti-hyperlipidemic, anti-hyperglycemic, anti-inflammatory and antioxidant properties [16, 17]. Interestingly, bona fide effects of hesperetin on lipid accumulation and adiposity have been previously demonstrated by various studies [18–21]. However, the role of hesperetin in the adipose tissue fibrosis has yet to be unraveled. Therefore, the objective of this study was to investigate the effect of hesperetin on Collagen VI and osteopontin as the major components of adipose tissue ECM, as well as main matrix metalloproteinases, Mmp2 and Mmp9, in 3T3-L1 pre-adipocytes.

## Materials and methods

### Chemicals and reagents

Hesperetin was obtained from Sigma-Aldrich (W431300, Germany). Cell culture reagents, including culture medium (L0093) and antibiotics (L0022) were obtained from Biowest (France). Fetal bovine serum (FBS) was purchased from Biosera (FB-1001/100, France). Hybrid-R RNA extraction kit (305 − 101, GeneAll Biotechnology, Korea) was used for the isolation of total cellular RNA and the corresponding cDNA was synthesized with HyperScript™ RT Master Mix (601–710, GeneAll Biotechnology, Korea). RealQ Plus 2x SYBR Green Master Mix high ROX™ (A325402, Ampliqon, Denmark) was used for real-time PCR. BCA Protein Assay Kit (23,225) was purchased from Thermo Fisher Scientific, UK). RIPA cell lysis buffer (PL008-5X) was attained from Biobasic (Canada). All the chemicals, including MTT (M5655), dimethyl sulfoxide (DMSO) (67-68-5), and general laboratory reagents were obtained from Sigma-Aldrich (Germany).

### Cell culture

3T3-L1 cells were purchased from Cell Bank of the Iranian Biological Resource Center (Tehran, Iran) and maintained in DMEM (Dulbecco’s modified Eagle’s medium), containing 10% FBS and 1% penicillin/streptomycin at 37 °C with 5% CO_2_. Cells were treated with different concentrations of hesperetin, after solubilization in DMSO, considering the final concentration of DMSO to be less than 0.1%. The highest concentration of DMSO in the treatments was also added as the negative control.

### Cell viability test

The viability of 3T3-L1 cells was evaluated using MTT assay. Cells were seeded in a 96-well plate at a density of 4 × 10^3^ cells/well. Then the cells were treated with various concentrations of hesperetin, including 25, 50, 100, 200, 300, 400, 500, 600, 700 and 800 µM for 24, 48 and 72 h. After incubation, the media was replaced with media containing 10 µl of MTT (stock concentration-5 mg/mL in PBS), followed by 3 h of incubation at 37℃ until formazan crystals were formed. The reaction was stopped by adding 100 µl DMSO to each well. After incubating in a dark place, at room temperature for 10 min, the absorbance was measured at 570 nm wavelength using a plate-reader. Cell viability was then calculated relative to control untreated cells.

### RNA isolation and real-time PCR

Total RNA was extracted from 3T3-L1 cells and the purity of RNA was measured with NanoDrop 2000 spectrophotometer (Thermo Fisher Scientific, USA). Reverse transcription was performed using 1 µg total RNA and real-time PCR was done in triplicate with SYBR green method, using an initial denaturation step (15 min at 95℃) and the subsequent 40 cycles of 30 s at 95℃ and 1 min at 61 °C. Beta-actin was used as the housekeeping gene and the relative gene expression was calculated by the 2^−ΔΔCt^ formula. The primer sequences are presented in Table 1.

**Table 1:** The information of primers used for real-time PCR.

Gene name	Gene acronym	Accession No.	Sequence	Product length
collagen type VI	*Col6a3*	XM_030245451.2	5’- AACCCTCCACATACTGCTAATTC-3’ 5’- TCGTTGTCACTGGCTTCATT-3’	70
Osteopontin (secreted phosphoprotein 1)	*Opn (Spp1)*	NM_001204203.1	5’- GCCTGTTTGGCATTGCCTCCTC-3’ 5’- CACAGCATTCTGTGGCGCAAGG-3’	158
matrix metallopeptidase 2	*Mmp2*	XM_006530751.4	5’- CAGGGAATGAGTACTGGGTCTATT-3’ 5’- ACTCCAGTTAAAGGCAGCATCTAC-3’	119
matrix metallopeptidase 9	*Mmp9*	NM_013599.5	5’- AATCTCTTCTAGAGACTGGGAAGGAG-3’ 5’- AGCTGATTGACTAAAGTAGCTGGA-3’	128
Beta-actin	*Actb*	NM_007393.5	5’- GTCCTCCTGGCATACCATAGA-3’ 5’- AGCTCAGTAACAGTCCGCCTAGA-3’	101

The species of all the primers are *Mus musculus*

### Western blotting

Total protein extraction and immunoblotting was performed as described previously [22]. In brief, 3T3-L1 cells were washed with ice-cold PBS, homogenized and lysed in lysis buffer containing protease and phosphatase inhibitor cocktail (Sigma-Aldrich, Germany). Protein concentrations were measured by BCA method, using bovine serum albumin as the reference standard. Total proteins (40 µg per well) were separated by 8% sodium dodecyl sulfate (SDS) polyacrylamide gel electrophoresis and transferred to polyvinylidene difluoride (PVDF) membranes (03010040001, Roche Applied Sciences, Germany). The membranes were incubated for 4 h at room temperature with 0.1% Tween 20 in tris-buffered saline (TBST) containing 5% skim milk for osteopontin and and 5% BSA for collagen VI determination, respectively. Subsequently, blots were washed and incubated overnight at 4 °C with buffer containing primary antibodies; either 1:1000 dilution of antibody against mouse collagen VI (Col6A1) (B-4, sc-377,143), 1:500 dilution of antibody against mouse osteopontin (AKm2A1, sc-21,742) (Santa Cruze Biotechnology, Inc., USA), or antibody against mouse GAPDH (6C5, ab-8245, Abcam, USA). Membranes were rinsed three times with TBST before and after the incubation with the secondary antibody for 1.5 h, at room temperature. Horseradish peroxidase-conjugated anti-mouse IgG Fc binding protein (sc-525,409, Santa Cruze Biotechnology, Inc., USA) was used as the secondary antibody. Immunodetection was performed using enhanced chemiluminescent (ECL) detection reagent (Amersham biosciences, UK) and the bands were detected through exposing blots to X-ray films. Quantification of the visualized bands was carried out using Image J software (NIH, Bethesda, USA).

### Statistical analysis

Statistical analysis was carried out with the aid of GraphPad Prism 5 software (San Diego, USA). All obtained data were expressed as mean ± SEM and analyzed by one-way analysis of variance (ANOVA), with Dunnett’s post-hoc test. The cutoff for significance was considered p < 0.05.

## Results

### The effect of hesperetin on the viability of 3T3-L1 pre-adipocytes

**Fig. 1:**
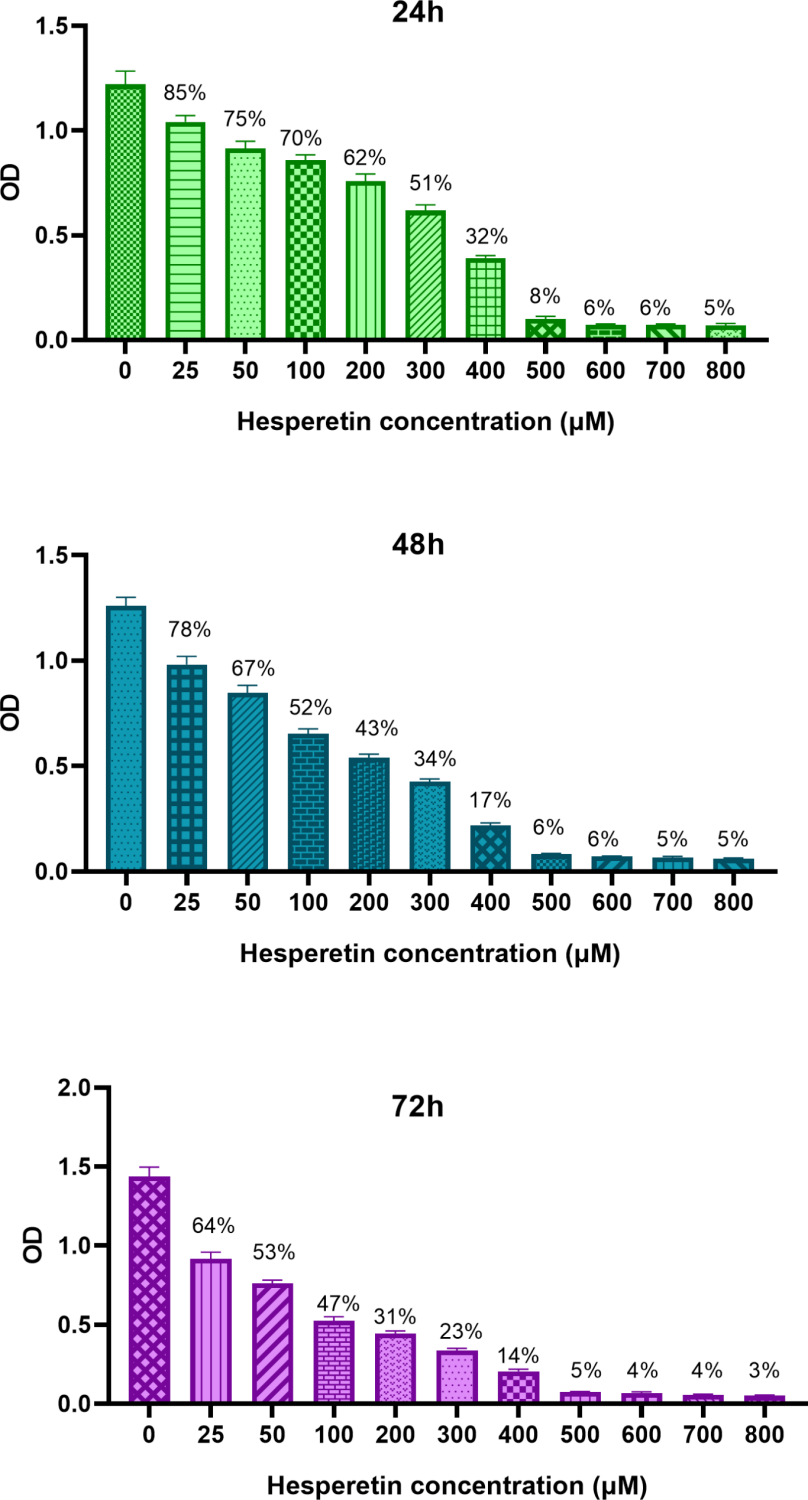
The viability of 3T3-L1 cells after 24 h, 48 h and 72 h treatment with various concentrations of hesperetin (25–800 µM), evaluated by MTT assay. The obtained results are compared with untreated control and presented as mean ± SD of at least three replicates

To investigate the cytotoxic effect of hesperetin on 3T3-L1 cells, first we examined cell viability in response to various concentrations of hesperetin. As shown in Fig. 1, the IC50 was found to be 447.4 µM, 339.2 µM and 258.8 µM for 24 h, 48 h and 72 h of treatment, respectively. Considering these data, we selected lower concentrations of hesperetin (25, 100 and 150 µM) for further experiments.

### Hesperetin reduced gene expression of ***Col6a3*** and ***OPN*** in 3T3-L1 pre-adipocytes

**Fig. 2:**
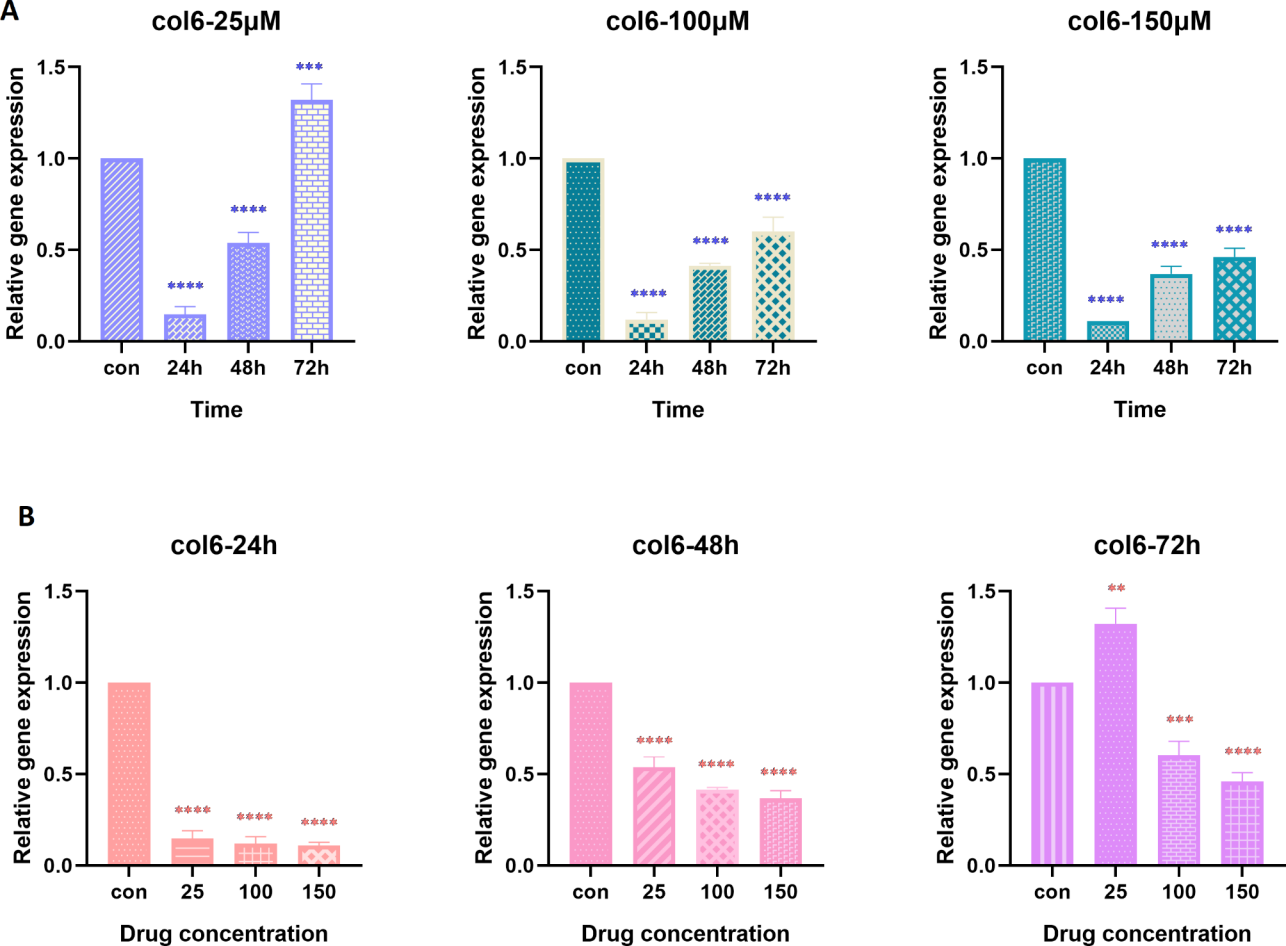
The time-dependent (**A**) and dose-dependent (**B**) effect of hesperetin on the expression of *Col6* gene, encoding Collagen VI, in 3T3-L1 pre-adipocytes. The controls in panel A are mean of the controls in 24 h, 48 h, and 72 h. The obtained results are presented as mean ± SD of at least three replicates. * p < 0.05, ** p < 0.01, *** p < 0.001, ****p < 0.0001

The expression of genes that encode coallagen VI *(Col6a3)* and osteopontin (*Opn)* was analyzed by real-time PCR technique. Figure 2 shows the effect of hesperetin on *Col6a3* gene expression in 3T3-L1 pre-adipocytes. Hesperetin could reduce *Col6a3* gene expression in a time- and dose-dependent manner. Moreover, we observed that the most significant effects of hesperetin on *Col6a3* gene expression was evident at 48 h and 72 h of treatment with all selected hesperetin concentrations (p value < 0.0001).

**Fig. 3:**
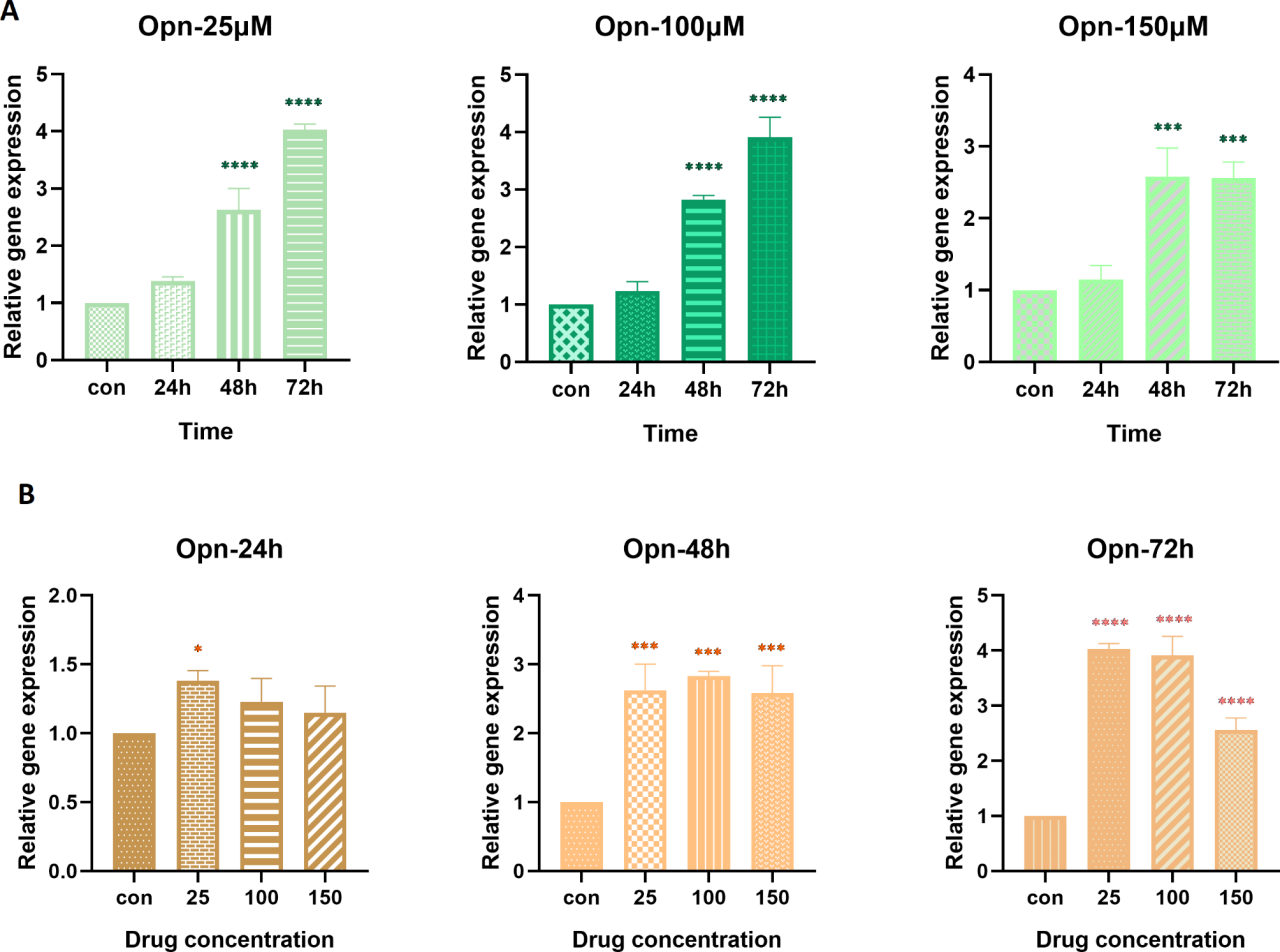
The time-dependent (**A**) and dose-dependent (**B**) effect of hesperetin on the expression of *Opn* gene, encoding osteopontin, in 3T3-L1 pre-adipocytes. The controls in panel A are mean of the controls in 24 h, 48 h, and 72 h. The obtained results are presented as mean ± SD of at least three replicates. *** p < 0.001, ****p < 0.0001

The effect of selected concentrations of hesperetin on *Opn* gene expression is illustrated in Fig. 3. From the presented results it is apparent that hesperetin led to a significant time- and dose-dependent decrease in the expression of *Opn* gene (p value < 0.0001).

### Hesperetin decreases protein level of collagen VI and osteopontin in 3T3-L1 pre-adipocytes

**Fig. 4:**
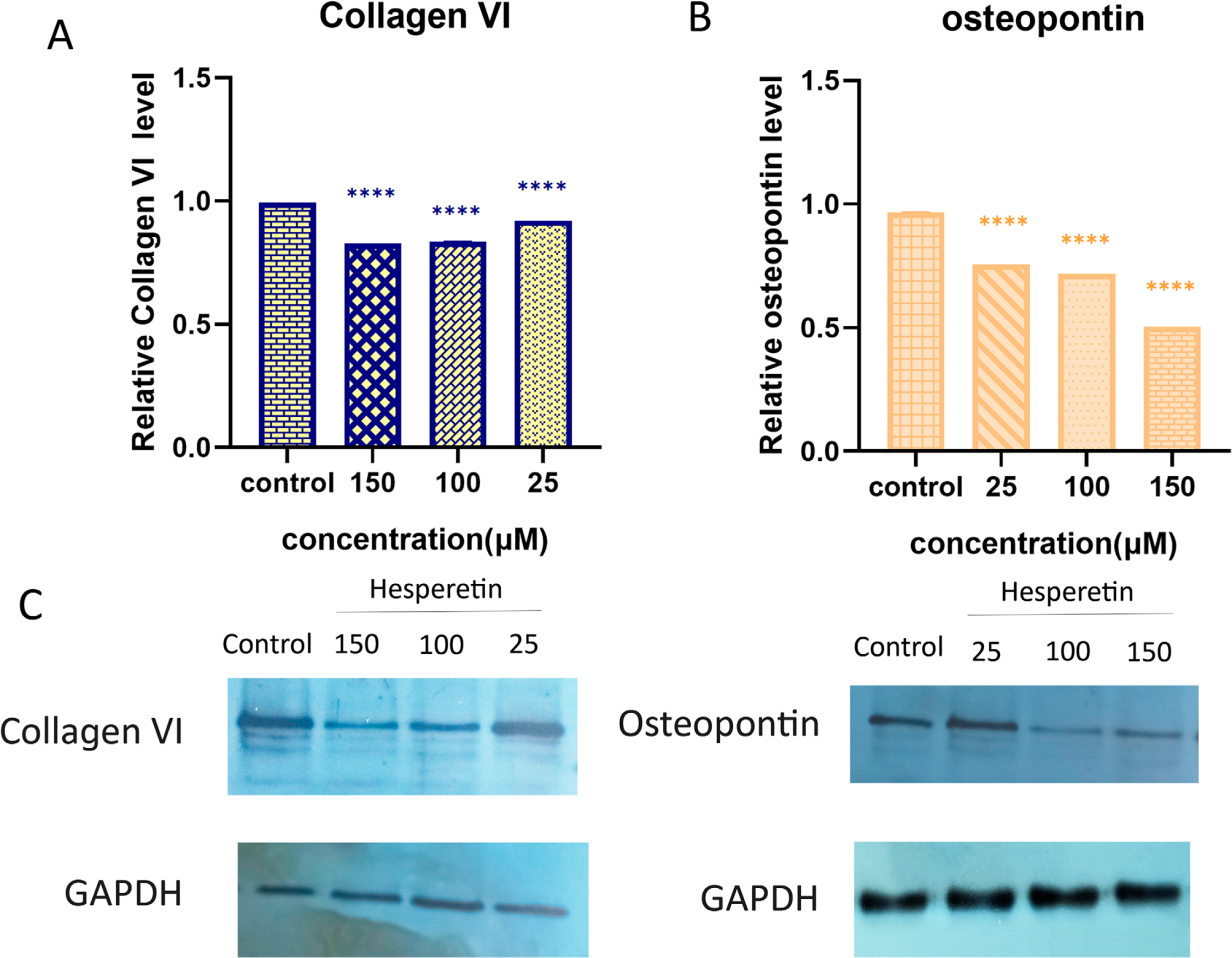
The effect of hesperetin on (**A**) collagen VI and (**B**) osteopontin protein levels in 3T3-L1 cells, compared to untreated control. (**C**) a representative Western blotting result is shown. Data are presented as mean ± SD of at least three replicates, ** P < 0.01, ****p < 0.0001

In order to further confirm the suppressive impact of hesperetin on collagen VI and osteopontin protein expression in pre-adipocytes, we conducted western blotting. From the data in Fig. 4, it can be seen that there was a remarkable decrease in the levels of both collagen VI and osteopontin proteins (p value < 0.0001) in response to treatment with hesperetin. Nevertheless, low dose of hesperetin (25 µM) did not cause a significant change in osteopontin protein level.

### Hesperetin reduced the gene expression of Mmp2 and Mmp9 in 3T3-L1 pre-adipocytes

**Fig. 5:**
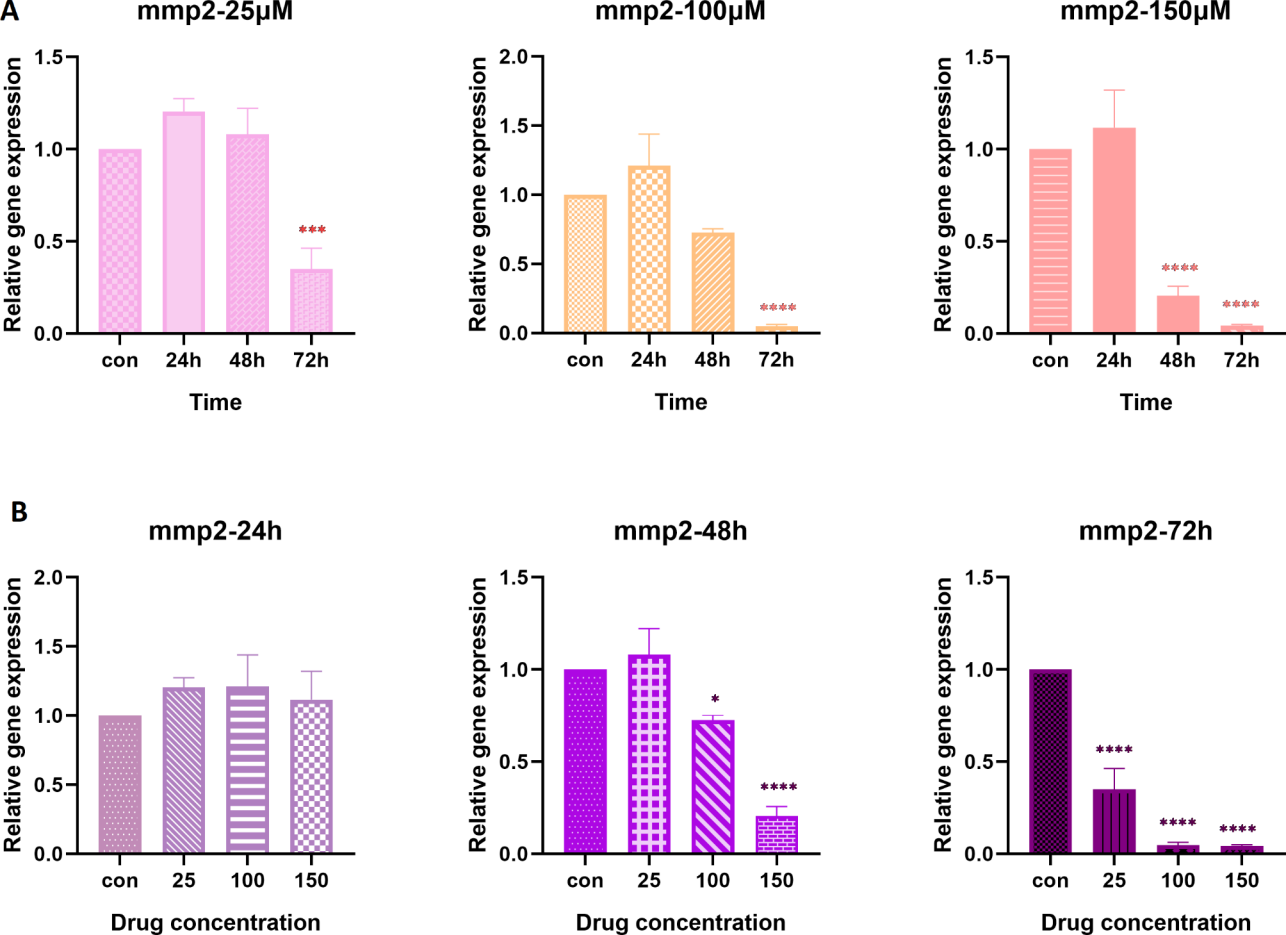
The effect of hesperetin on the gene expression of *Mmp2* in different times (**A**) and concentrations (**B**). The controls in panel A are the mean of the control in 24 h, 48 h, and 72 h. The obtained results are presented as mean ± SD of at least three replicates. ** p < 0.01, *** p < 0.001, ****p < 0.0001

Amongst the matrix metalloproteinase enzymes, Mmp-2 and Mmp-9 are the main contributors to adipose tissue fibrosis, the levels of which are noticeably surged in response to the accumulated ECM components [23, 24]. With regard to the inhibitory effect of hesperetin on collagen VI and osteopontin as major ECM proteins, we were keen to investigate the expression level of *Mmp2* and *Mmp9* after hesperetin exposure. As it is shown in Fig. 5 there is a clear trend of decreasing *Mmp2* gene expression in a time- and dose-dependent manner as compared to untreated control cells. Data showed that *Mmp2* gene expression was significantly decreased following treatment with almost all concentrations of hesperetin and different time spans (*p value < 0.0001*). However, treatment with 25 µM hesperetin resulted in no statistically significant change in *Mmp2* gene expression.

**Fig. 6:**
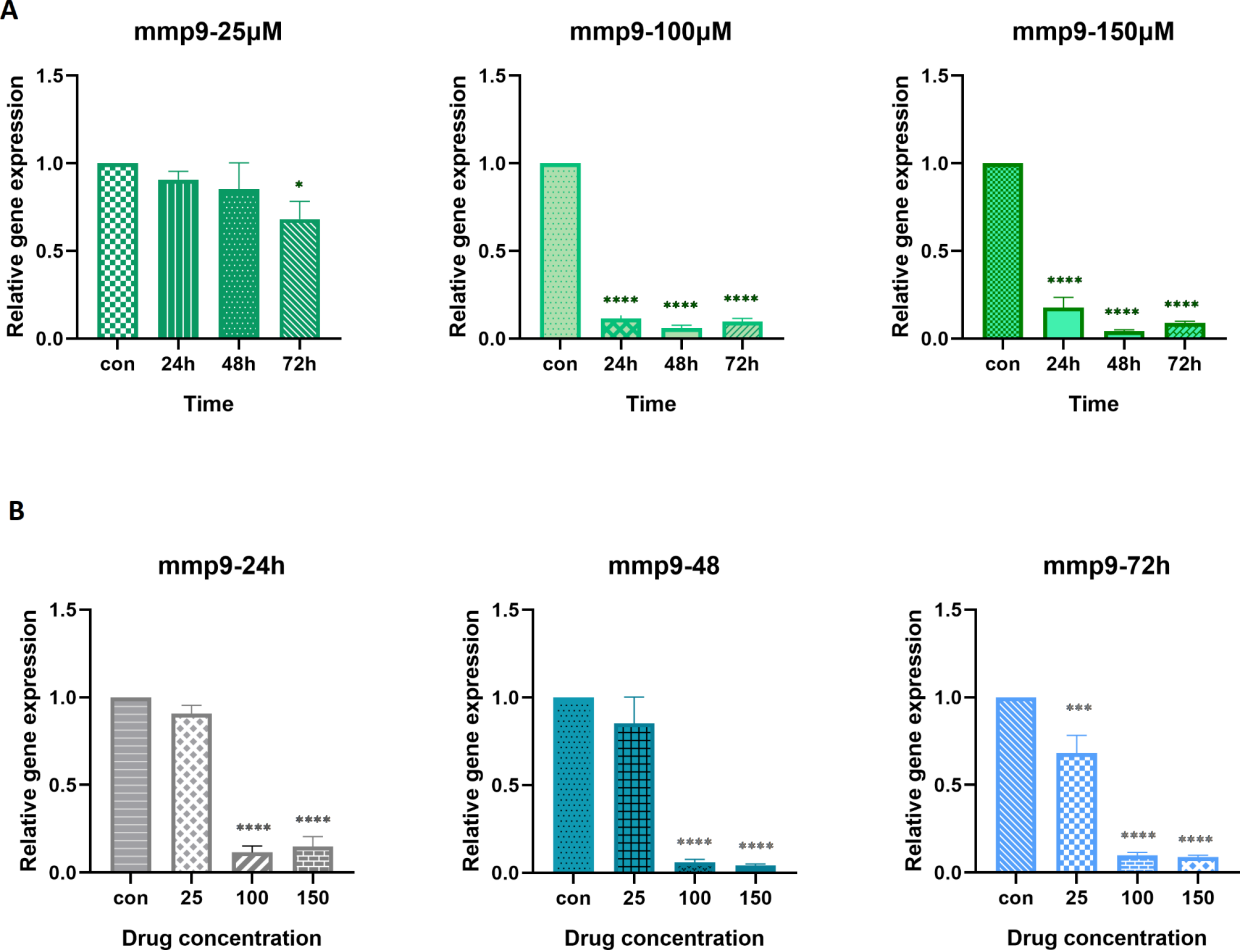
The effect of hesperetin on the gene expression of *Mmp9* in different times (**A**) and concentrations (**B**). The controls in panel A are the mean of the controls in 24 h, 48 h, and 72 h. The obtained results are presented as mean ± SD of at least three replicates. * p < 0.05, *** p < 0.001, ****p < 0.0001

Hesperetin could also effectively decrease *Mmp9* gene expression in almost all doses and time spans in comparison to control cells (Fig. 6). Although 25 µM hesperetin caused no significant change on the *Mmp-9* gene expression after 24 and 48 h of treatment. Furthermore, it was found that there was no notable difference between 100 µM and 150 µM concentrations of hesperetin at different time intervals.

## Discussion

Many studies have examined the effect of hesperetin on obesity and its complications [25–28]. Citrus flavonoids, including hesperetin were shown to suppress gene expression of stearoyl-CoA desaturase, an enzyme whose inhibition reduces hyperlipidemia and adiposity [29]. Additionally lipolytic actions of tumor necrosis factor α (TNF-α), which promotes insulin resistance, can be attenuated by hesperetin in 3T3-L1 cells [28]. Intriguingly, hesperetin and hesperidin have the ability to stimulate the release of one of the appetite-regulating hormones, named cholecystokinin (CCK), suggesting that hesperetin might serve as a candidate biomolecule for the suppression of appetite and weight gain [30].

Herein, we focused on the anti-fibrotic properties of hesperetin and thus, aimed to find its impact on the major ECM constituents (collagen VI and osteopontin). Adipose tissue ECM is a complex and rich meshwork of dynamically changing interconnected macromolecules, of which collagen VI and osteopontin are among the main constituents [31]. It has been previously shown that in human body, expression of collagen VI and osteopontin increases during obesity [32, 33]. Khan et al., demonstrated an inverse correlation between collagen VI deposition in adipocyte-surrounding ECM and insulin sensitivity as well as inflammatory phenotype of obesity [10]. Osteopontin has also been shown to be expressed in adipose tissue and the associated macrophages, and promotes inflammation [34]. A myriad of studies have proposed osteopontin as an important adipokine, playing a key role in linking obesity to insulin resistance through adipose tissue macrophage recruitment [13, 33]. We had also previously shown that visfatin, an inflammatory adipocytokines, can promote fibrosis through modulation of the expression of ECM components [22]. However, attenuation of adipose tissue fibrosis by natural products is a topic which has not been scrutinized well.

Our results in the present study showed for the first time that hesperetin could remarkably reduce collagen VI and osteopontin at protein and mRNA levels in 3T3-L1 pre-adipocytes. A major hallmark of white adipose tissue fibrosis is the accumulation of ECM proteins, which increases the risk of insulin resistance [35, 36]. Hence, hesperetin-mediated downregulation of collagen VI and osteopontin might be considered as an anti-fibrotic property. The effect of hesperetin on the ECM components has not been previously investigated in pre-adipocytes; however, consistent with our findings, it has shown to be effective in the amelioration of liver fibrosis in different hepatocyte injury models. For example, hesperetin has been reported to exert protective effects against CCl_4_-induced liver fibrosis in animal models [37, 38], as well as high-fat diet-induced non-alcoholic fatty liver disease [39]. Furthermore, the anti-fibrotic influence of hesperetins has been demonstrated in experimental hepatocytes [39, 40]. These studies have mainly focused on anti-oxidative and anti-inflammatory properties of hesperetin; however, down-regulation of Col1α1, Col3α1 and TIMP-1 in hepatocytes has been reported [38, 40]. The exact molecular mechanism behind the protective effect of hesperetin in fibrosis is not well-defined, but the activation of AMPK/SIRT3, suppression of Glioma associated oncogene-1 (Gli-1), increased expression of Nrf2, and attenuation of the aberrant expression of patched1 in the Hedgehog pathway have been proposed as underlying mechanisms in the amelioration of liver fibrosis [37, 38, 40, 41].

Not many herbal derivatives have been investigated in relation to the markers of ECM accumulation in adipocytes; nevertheless, the scarce available research shows the ability of these compounds to combat adipose tissue fibrosis. Saponins derived from Panax japonicus plant was shown to decrease collagen deposition and suppress the expression of genes involved in fibrosis in epididymal white adipose tissue of obese mice [42]. In another study by Wang et al., barberine was shown capable of attenuating adipose tissue fibrosis in mice model of obesity [43]. Isoliquiritigenin, a flavonoid derived from *Glycyrrhiza uralensis* plant, improves fibrosis in adipose tissue though inhibition of TLR4- and Mincle‐induced expression of fibrosis‐related genes in adipocytes and macrophages [44].

The matrix metalloproteinases are fundamental enzymes in ECM homeostasis, playing a major role in ECM degradation [45]. Increased levels of MMP-2 and MMP-9 in subjects with obesity and type 2 diabetes, as well as obesity-related insulin resistance have been demonstrated in several studies [22, 45, 46]. Here, we showed that *Mmp2* and *Mmp9* mRNA levels were decreased in 3T3-L1 cells treated with hesperetin. These findings are consistent with previous studies performed in tumor cells, which reported that hesperetin is capable of suppressing both MMP-2 and MMP-9 [47, 48]. These data further support that hesperetin not only reduces ECM proteins but also suppresses the expression of MMP enzymes.

The limitation of this study was that we did not include animal model of obesity and therefore could not evaluate adipose tissue fibrosis at the tissue levels. However, given the potency of the hesperetin in the modulation of crucial markers of fibrosis, animal studies to confirm these beneficial effects, especially long-term influences on insulin resistance, would be plausible to consider for future studies. Since repression of adipose tissue fibrosis improves systemic glucose homeostasis independent of body-weight loss [9], remedies to alleviate this condition would be highly beneficial in the reduction of obesity-associated complications.

## Conclusion

In conclusion, the evidence from this study reveals the benefit of hesperetin against the pathophysiology of adipose tissue fibrosis, through downregulation of ECM proteins and major MMPs. The efficacy of hesperetin in reducing the markers of fibrosis introduces this compound as a probable anti-fibrotic therapeutic option in obesity and therefore, hesperetin might be considered as a solution to improve obesity-associated metabolic disorders.

### Electronic supplementary material

Below is the link to the electronic supplementary material.

Supplementary Material 1

## Data Availability

Data will be made available from corresponding author (Mitra Nourbakhsh, nourbakhsh.m@iums.ac.ir) upon reasonable request.

## Abbreviations

ECM: extracellular matrix
Mmp: matrix metalloproteinase
T2DM: type 2 diabetes mellitus
NAFLD: non-alcoholic fatty liver disease
NASH: non-alcoholic steatohepatitis
DMSO: dimethyl sulfoxide
SDS: sodium dodecyl sulfate
PVDF: polyvinylidene difluoride
TBST: Tween 20 in tris-buffered saline
ECL: enhanced chemiluminescent
ANOVA: one-way analysis of variance
CCK: cholecystokinin
TNF-α: tumor necrosis factorα
