# Supplementary figures and images for: Hesperetin attenuates the expression of markers of adipose tissue fibrosis in pre-adipocytes
Source: BMC Complement Med Ther. 2023 Sep 11;23:315. doi: 10.1186/s12906-023-04152-z (PMC10496229; doi:10.1186/s12906-023-04152-z)

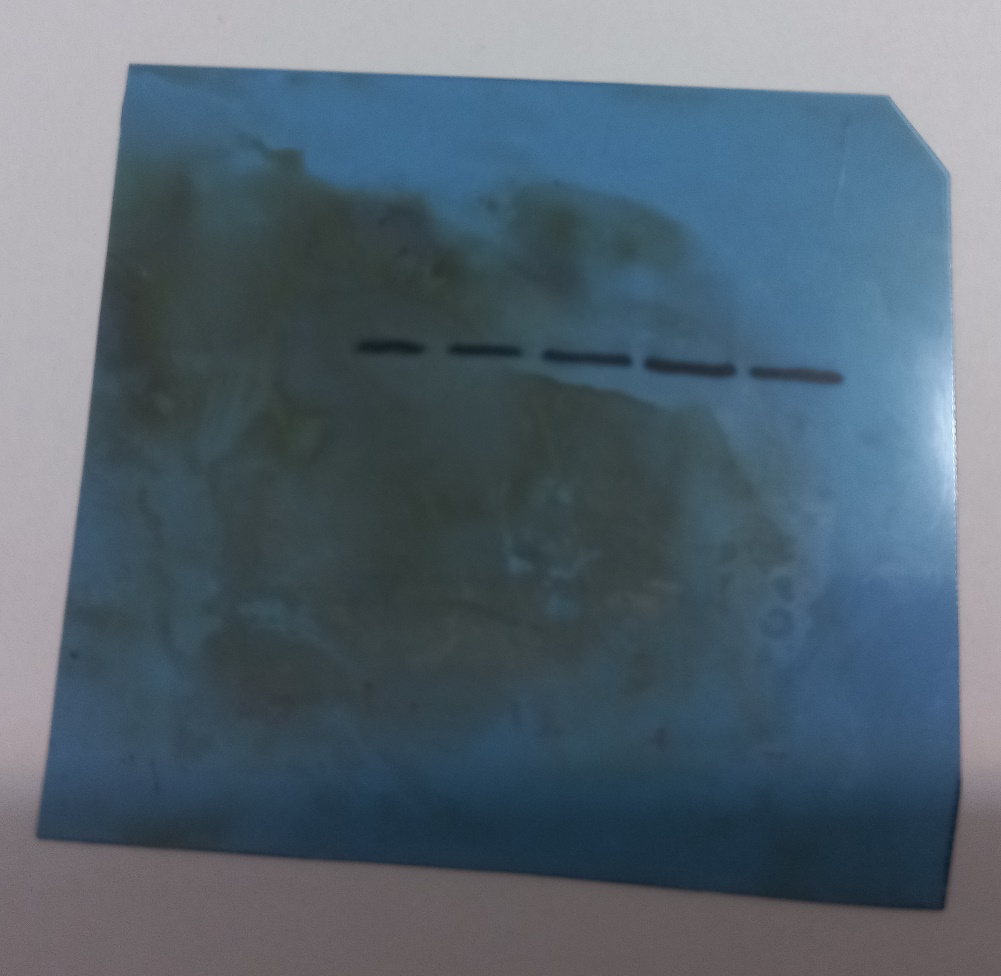


**GAPDH**

**Collagen VI**


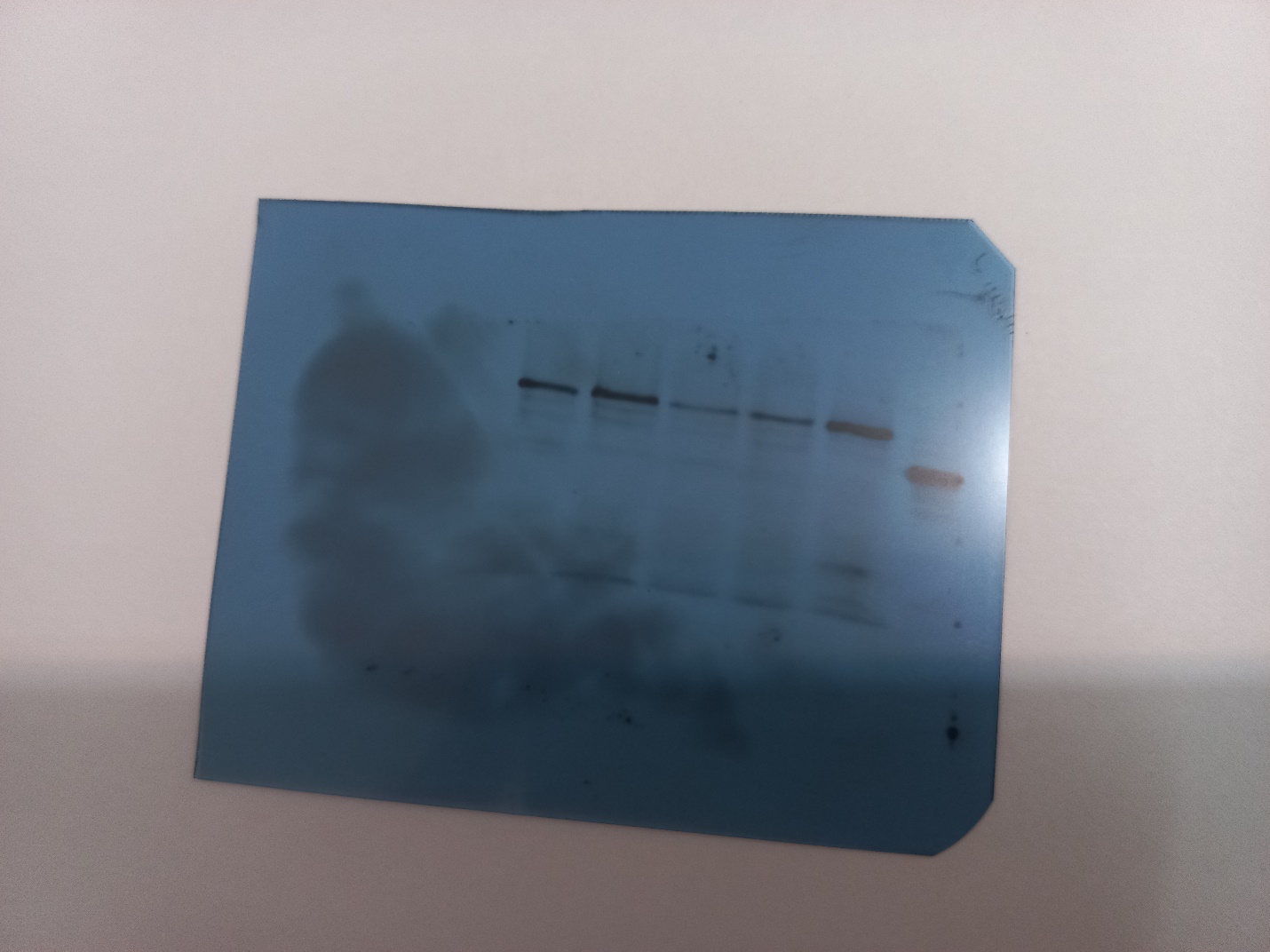


**Osteopontin**


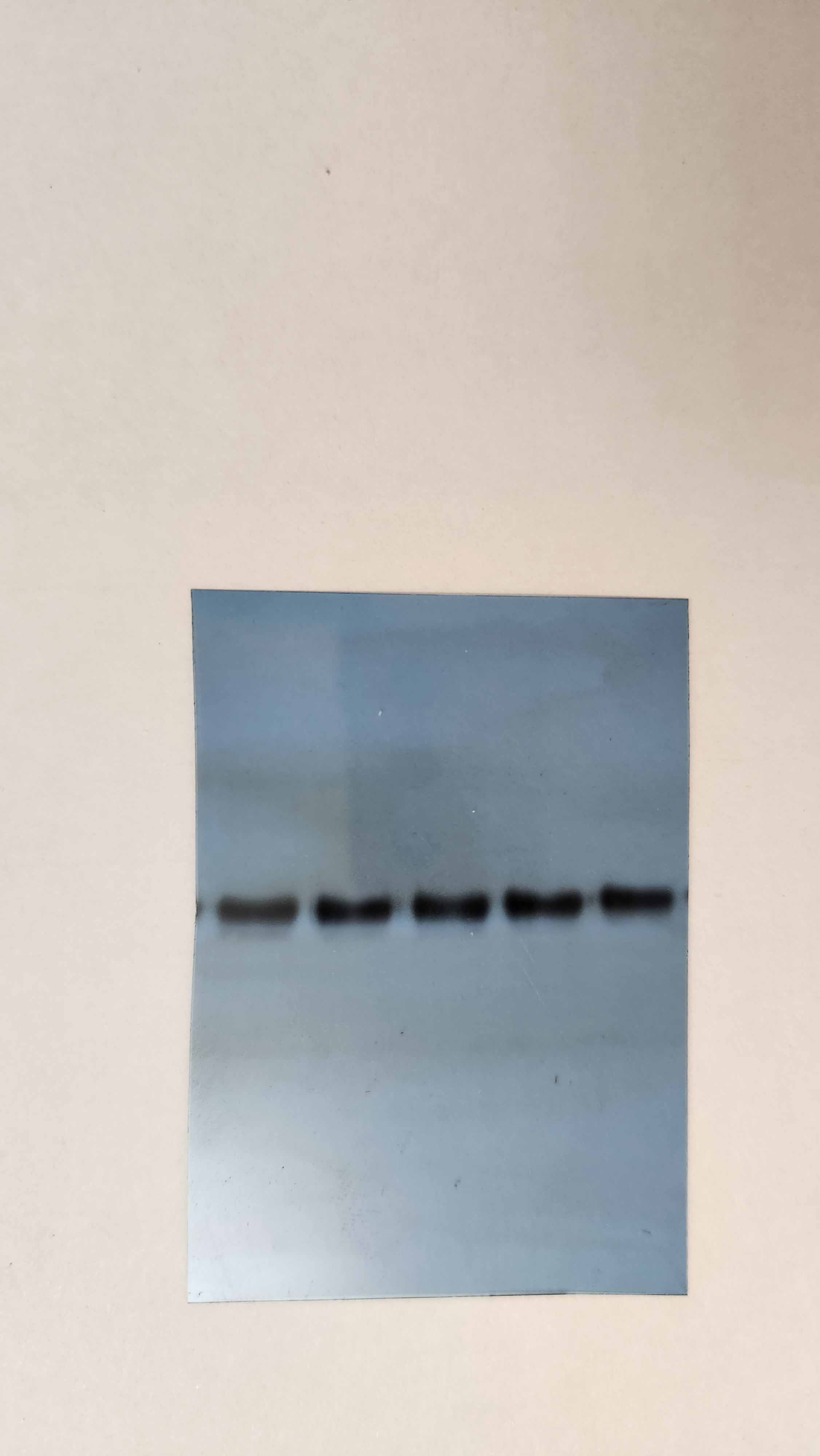


**GAPDH**

**Osteopontin**

**Collagen VI**

Supplement: Supplementary file 1 — Supplementary Material 1 [file 12906_2023_4152_MOESM1_ESM.docx]
